# Neem Leaf Glycoprotein Prophylaxis Transduces Immune Dependent Stop Signal for Tumor Angiogenic Switch within Tumor Microenvironment

**DOI:** 10.1371/journal.pone.0110040

**Published:** 2014-11-12

**Authors:** Saptak Banerjee, Tithi Ghosh, Subhasis Barik, Arnab Das, Sarbari Ghosh, Avishek Bhuniya, Anamika Bose, Rathindranath Baral

**Affiliations:** Department of Immunoregulation and Immunodiagnostics, Chittaranjan National Cancer Institute (CNCI), Kolkata, India; Jawaharlal Nehru University, India

## Abstract

We have reported that prophylactic as well as therapeutic administration of neem leaf glycoprotein (NLGP) induces significant restriction of solid tumor growth in mice. Here, we investigate whether the effect of such pretreatment (25µg/mice; weekly, 4 times) benefits regulation of tumor angiogenesis, an obligate factor for tumor progression. We show that NLGP pretreatment results in vascular normalization in melanoma and carcinoma bearing mice along with downregulation of CD31, VEGF and VEGFR2. NLGP pretreatment facilitates profound infiltration of CD8^+^ T cells within tumor parenchyma, which subsequently regulates VEGF-VEGFR2 signaling in CD31^+^ vascular endothelial cells to prevent aberrant neovascularization. Pericyte stabilization, VEGF dependent inhibition of VEC proliferation and subsequent vascular normalization are also experienced. Studies in immune compromised mice confirmed that these vascular and intratumoral changes in angiogenic profile are dependent upon active adoptive immunity particularly those mediated by CD8^+^ T cells. Accumulated evidences suggest that NLGP regulated immunomodulation is active in tumor growth restriction and normalization of tumor angiogenesis as well, thereby, signifying its clinical translation.

## Introduction

In 2000, Hanahan and Weinberg described angiogenesis as one of the most important hallmark criterion for cancer [1]. In spite of fundamental role of angiogenesis in fetal development and in many physiological conditions like wound healing [2], [3] tumors exploit it to promote blood vessel growth and fuel a tumor's transition from benign to a malignant state [4], [5]. Likewise, these malignant transformations need evasion from immune destruction, which has been included recently, in 2011, as another important hallmark of cancer growth [6]. Angiogenesis and immune evasion, these two apparently parallel cancer-intrinsic phenomenon actually possess bidirectional link and convergely promote malignant growth, metastasis and ultimately regulate therapeutic outcome [7]. In cancer, immune system can regulate angiogenesis with both pro- and anti-angiogenic activities [8], [9]. Angiogenic molecules by differentially regulating immune system help in the development of sustained immunosuppressive mechanisms within tumor microenvironment (TME) [10], [11]. This immunosuppressive mechanism may promote angiogenesis and tumor growth and inhibits infiltration and homing of activated immune cells within TME. Promoted angiogenesis then deregulates the proliferation and migration of vascular endothelial cells (VECs), thereby, causing neovascularization. These results in aberrant tumor vasculature associated with distorted and enlarged vessels, increased permeability, irregular blood flow and micro-hemorrhages [10], [12], [13]. Therefore, in recent years different works have shown that, for optimum immune-mediated tumor destruction, normalization of tumor vasculature is preferred over complete blockade of tumor angiogenesis [14].

Neem leaf glycoprotein (NLGP), a nontoxic immunomodulator reported previously have significant murine tumor growth restricting potential in prophylactic [15], [16] as well as therapeutic [17], [18] settings. NLGP facilitates anti-tumor activity by modulating both systemic and local immunity including: i) suppression of regulatory T cells [19], ii) activation of effector NK, NKT and T cells [20], [21], iii) modulation of antigen presenting cells by maturating dendritic cells (DCs) towards DC1 phenotype [22], [23] and macrophages [24], iv) regulation of cytokine-chemokine balance [25], [26] and v) preventing anergy and exhaustion of effector T cells [17], [18]. Recently in two consecutive studies, we have reported that therapeutic effectiveness of NLGP is associated with profound tumor infiltration of CD8^+^ T cells [27] and normalization of tumor-immune-microenvironment [27], [28].

Therefore, in the present study, we prophylactically applied NLGP in murine carcinoma and melanoma bearing mice to boost antitumor immune responses and subsequently analyzed the mode of NLGP counteraction on the tumor angiogenesis. We report that NLGP pretreatment associated immune-stimulation, particularly CD8^+^ T cell activation, regulates the balance between pro- and anti-angiogenic molecules to induce vascular normalization without affecting normal physiological angiogenesis.

## Results

### NLGP prophylaxis prevents tumor angiogenesis and normalizes tumor vasculature

**Figure 1:**
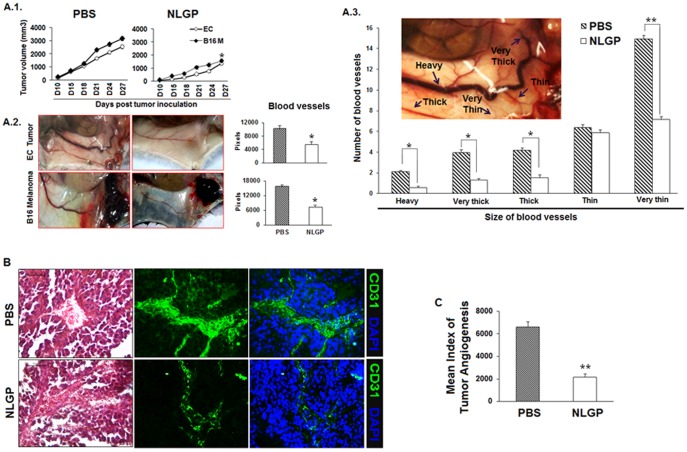
NLGP normalizes tumor vasculature. Swiss and C57BL/6 mice were pretreated with NLGP (25µg) once a week for four weeks in total followed by inoculation of EC (1×10^6^ cells/mice) and B16 melanoma cells (2×10^5^ cells/mice) subcutaneously. **A.1.** Tumor growth curve till day 27 is presented. *p<0.01. **A.2.** Mice were sacrificed and their angiogenic profile was studied and presented in photographs and bar diagrams (Mean±SD of pixel values). *p<0.01. **A.3.** Differentially dilated angiogenic vessels as shown in a representative figure (*inset*) were counted from NLGP and PBS treated mice and presented in bar diagram. *p<0.05; **p<0.001. **B.** Angiogenic blood vessels within tumors were studied by routine histology after H&E staining and CD31^+^ VECs were studied by immunofluorescence staining. Representative figures in each case are presented. **C.** Mean index of tumor angiogenesis is presented in bar diagram. **p<0.001.

‘Dormant’ tumor requires both angiogenic switch and immune escape to proceed towards malignancy [7], [9]. As prophylaxis with neem leaf preparation, precursor of NLGP, previously reported to be associated with significant immune-mediated tumor growth restriction [16], [29], here, we intended to study how NLGP prophylaxis regulates pathological tumor angiogenesis. Consistent with our previous results prophylactic NLGP administration (4×) significantly restricts Ehrlich's carcinoma and B16 melanoma tumor growth (Figure 1a). Repeated investigations confirmed 4 immunizations with NLGP are required for optimum immune activation [15]–[18], [27]–[29]. Angiogenic profiles were studied in mice after establishment of tumor (in between day 21 to 32) (Figure 1A.1 and A.2) and visual observations suggested a significant decrease in heavy, very thick, thick blood vessels, while thin blood vessels were retained substantially in NLGP pretreated carcinoma and melanoma tumor bearing mice group compared to PBS controls (Figure 1A.3). Additionally, histological analysis of tumor sections demonstrated less number of blood vessels with more regularized pattern in NLGP pretreated tumors than PBS mice. This regularized pattern of blood vessels is further evidenced by downregulation of CD31, a marker of VECs (Figure 1B). Correlating angiogenic profile with tumor volume revealed that normalized angiogenesis associated with NLGP prophylaxis represents restricted tumor growth, whereas, chaotic angiogenesis is correlated well with bigger tumor volume (Table 1; Figure 1C). Therefore, these data furnish evidences that NLGP can normalize tumor vasculature by decreasing only the thick and ‘tortured’ blood vessels while retaining the more compact thin blood vessels within tumor to maintain the optimum interstitial pressure and vaccine mediated immune benefits.

**Table 1:** MITA* relating tumor volume and angiogenesis in NLGP pretreated mice.

PBS			NLGP		
Tumor Volume (in mm^3^)	Angiogenesis (in raw score)	Index for Tumor Angiogenesis	Tumor Volume (in mm^3^)	Angiogenesis (in raw score)	Index for Tumor Angiogenesis
220	+ (1)	220	126	+ (1)	126
600	++ (2)	1200	245	+ (1)	245
907	++ (2)	1814	665	++ (2)	1330
2025	++++ (4)	8100	1267	+++ (3)	3801
1152	+++ (3)	3456	445	+ (1)	445
3240	++++ (4)	12960	1568	++ (2)	3136
5292	++++ (4)	21168	1436	+++ (3)	4308
1352	+++ (3)	4056	1008	++ (2)	2016
**Mean**		**6622**			**1926**

*Mean index of tumor angiogenesis. Mean is presented in Figure 1C

### NLGP mediated vascular normalization is associated with down regulation of CD31, VEGF and VEGFR2

**Figure 2:**
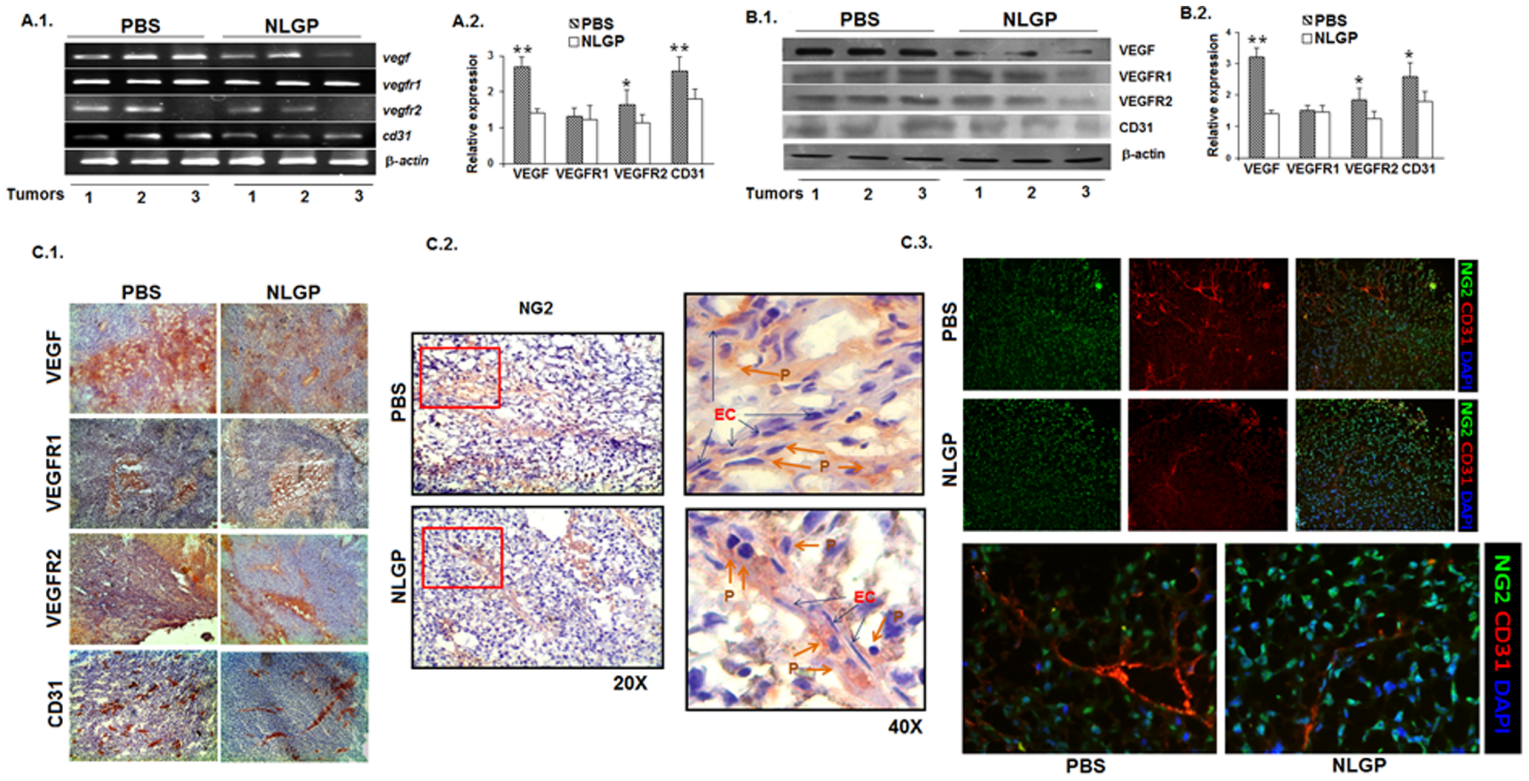
NLGP normalizes tumor microenvironment by downregulating expression of VEGF, VEGFR2 and CD31. B16 melanoma tumors (100 mm^3^) harvested from either PBS or NLGP pretreated C57BL/6 mice representing tumor microenvironment were used for different analysis. **A.1.** Representative presentation of mRNA expression levels of *vegf, vegfr1, vegfr2, cd31* by RT-PCR analysis (n = 3). **A.2.** Densitometric analysis of three individual observations is presented with Mean ± SD. *p<0.05; **p<0.01. **B.1.** Another portion (100 mg) of tumors was lysed by freeze-thaw cycles in PBS used for Western Blotting to check the expression of various angiogenic proteins. Representative presentation of expression levels of different molecules as mentioned by Western blot analysis (n = 3) is shown. **B.2.** Densitometric analysis of three individual observations and Mean ± SD are presented. *p<0.05; **p<0.01. **C.1.** Immunohistochemistry with monoclonal antibodies, specific for VEGF, VEGFR1, VEGFR2 and CD31 and **C.2.** NG2 were detected on tumor sections. Arrows showed the pericyte coverage on endothelial cell lining on blood vessels. **D.** Fluorescence tagged monoclonal antibodies, specific for NG2^+^ (green) and CD31^+^ (Red) cells were used to study tumor vasculature. Nuclear staining was performed by DAPI.

As VEGF-VEGFR2 signaling axis represents the key event in promoting tumor angiogenesis [30]–[32], expression of these molecules along with other pro-angiogenic molecules were next analyzed in NLGP pretreated carcinoma and melanoma bearing mice. Evidences obtained from RT-PCR (Figure 2A.1 and A.2)and Western Blot analyses demonstrated downregulation of VEGF, VEGFR2, CD31 in tumor from NLGP pretreated mice (Figure 2B.1 and B.2), in comparison to tumor obtained from PBS treated mice. Consistently, immunohistochemical analysis also revealed the significant decrease in expression level of VEGF and its receptor, VEGFR2, along with endothelial cell associated protein CD31 in harvested tumors with NLGP pre-therapy (Figure 2C.1). However, minimal decrease in VEGFR1 and NG2 level (Figure 2C.1 and C.2) was observed with similar treatment. Dual immunofluorescence staining of CD31 with NG2 (Figure 2C.3) indicated optimum and close pericyte coverage over VECs that may help in stabilization of blood vessels in NLGP treated mice group while, in PBS treated mice NG2^+^ pericytes were found detached from endothelial cells (Figure 2C.2), rendering the blood vessels thick, dilated and leaky. Therefore, obtained results clearly suggest that NLGP mediated alteration of pericytes' nature and/or that attachment along the vessel wall is intimately associated with observed vascular normalization within tumor.

### NLGP mediated vascular normalization requires host's intact immune-system

**Figure 3:**
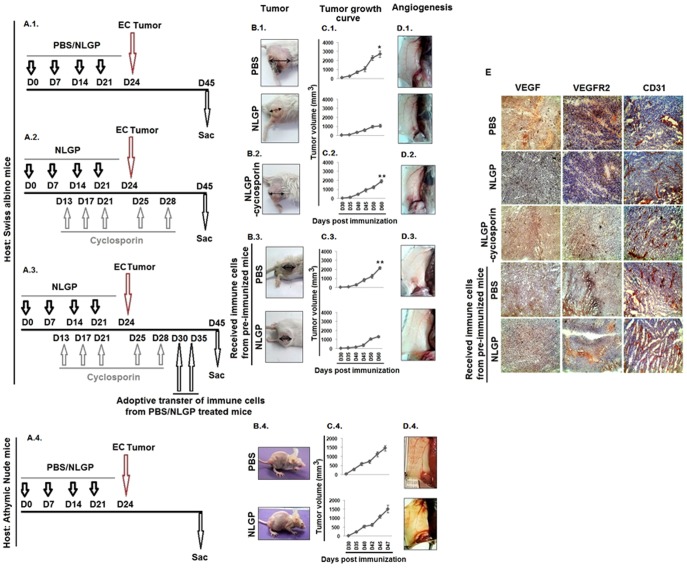
NLGP mediated normalization of angiogenesis is absent in immunocompromised mice. Schematic presentation of NLGP prophylaxis in **A.1.** normal mice, **A.2.** and **A.3.** cyclosporine treated/immunocompromised and **A.4.** athymic nude mice. **B.1.-B.4.** Representative photographs of murine tumors with **B.1.** PBS, NLGP, **B.2.** NLGP-Cyclosporin pretreatment, **B.3.** NLGP pretreatment with adoptive transfer of immune cells and **B.4.** NLGP and PBS pretreatment (athymic nude mice). **C.1.-C.4.** Tumor growth curve presenting Mean ± SD. *p<0.001, **p<0.01, in comparison to NLGP group with other above mentioned group and **D.1.-D.4.** Angiogenic profile of mice with **D.1.** PBS, NLGP, **D.2.** NLGP-Cyclosporin pretreatment, **D.3.** NLGP pretreatment with adoptive transfer of immune cells and **D.4.** NLGP and PBS pretreatment (athymic nude mice). **E.** Immunohistochemical detection of VEGF, VEGFR2 and CD31 in tumor sections as mentioned in **A.1–A.3**.

Several recent studies have demonstrated that angiogenesis and suppressed cell-mediated immunity interdependently play central role in the pathogenesis of malignant disease facilitating tumor growth [33], [34]. As NLGP prophylaxis reciprocally regulate tumor immune surveillance and angiogenesis to restrict murine tumor growth, next, we used two types of mice models (drug-induced immunosuppressive mice and immunocompromised athymic nude mice) to assess the immune involvement in NLGP mediated angiogenic modulation. As shown in (Figure 3A.1, A.2), mice were divided into three groups and two groups were injected with NLGP prophylactically, while one group was retained as control. Between these two NLGP pretreated mice groups, one group received immunosuppressant cyclosporine before EC tumor challenge as mentioned in ‘*Materials and Methods’*. Analysis of their angiogenic profile revealed that NLGP pretreatment caused significant normalization of tumor vasculature than control group, as demonstrated in Figure 3A.1 However, in cyclosporine group, NLGP pretreatment failed to normalize angiogenesis, and so they showed prominent with prominent dilated and fragile blood vessels (Figure 3A.1, A.2-D.1, D.2). Observed results clearly indicated that NLGP modulates angiogenesis or vascular normalization by activating immune system.

Corroborately, in a separate set of experiments, tumor growth and associated angiogenesis were studied in three groups of NLGP-cyclosporin treated EC bearing mice. Two such groups of mice adoptively received non-adherent immune cells from either NLGP or PBS immunized normal mice (Figure 3A.3). Mice from all the 3 groups were sacrificed after tumor reached a considerable volume (1500 mm^3^ to 2000 mm^3^ approximately) and their angiogenic profiles were analyzed (Figure 3B.3–D.3). Enhanced angiogenesis related to the cyclosporine mediated immunosuppression in NLGP pretreated mice was observed to be almost normalized due to adoptive transfer of splenic immune cells from NLGP treated mice (Figure 3B.3–D.3). These findings might further conclude that NLGP mediated normalization of angiogenesis is immune dependent.

To further validate the influence of NLGP-conditioned immune system to restrain tumor angiogenesis, immune-compromised athymic nude mice were pretreated with NLGP before tumor (EC) inoculation. However, NLGP pretreated nude mice failed to normalize tumor angiogenesis (Figure 3A.4–D.4) and adoptive transfer of syngenic non-adherent immune cells or isolated T cells of NLGP immunized normal mice showed tumor growth restriction and vascular normalization or inhibition in angiogenesis in comparison to PBS treated or only NLGP treated group *(data not shown).*

Tumors harvested from mice with different compromised immune systems with either pretreatment with NLGP or adoptive transfer of immune cells were analyzed for the expression status of VEGF, VEGFR2 and CD31, as we earlier found that NLGP downregulates the elevated expression levels of these molecules during tumor growth. Immunosuppression, either by means of cyclosporine treatment or in nude mice, abrogated the NLGP mediated downregulation of VEGF, VEGFR2 and CD31 (Figure 3E). Thus, NLGP may normalize angiogenesis by restricting availability of pro-angiogenic molecules.

### CD8^+^ T cells play vital role in NLGP mediated immune dependent vascular normalization

**Figure 4:**
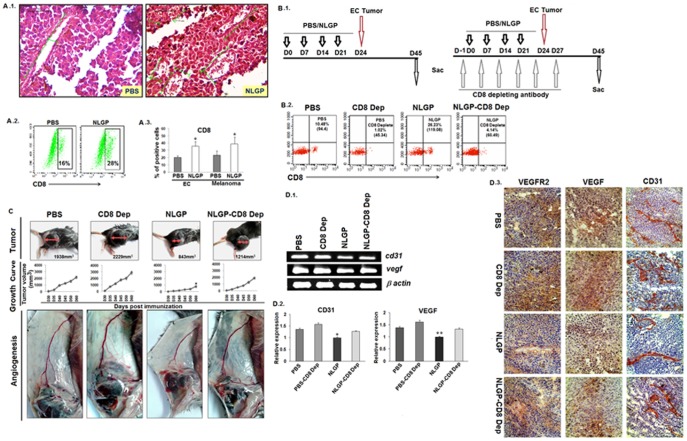
NLGP mediated normalization of tumor vasculature is dependent on CD8^+^ T cells. B16 melanoma tumors were harvested from both PBS and NLGP pretreated C57BL/6 mice. **A.1.** Immune infiltration within tumors from PBS and NLGP treated mice were assessed histologically (H&E). **A.2.** Status of CD8^+^ T cells in blood was assessed by flow cytometry. **A.3.** Bar diagram shows the status of CD8^+^ T cells within carcinoma and melanoma tumors. *p<0.01. **B.1.** Schematic presentation of control and CD8^+^ T cell depletion in either PBS or NLGP pretreated mice. **B.2.** Status of CD8^+^ T cells in all four mice groups (PBS, NLGP, PBS-CD8 dep and NLGP CD8 dep) were presented with representative figures. **C.** Representative picture of tumors, tumor growth curve and angiogenesis of PBS and NLGP pretreated mice with or without CD8^+^ T cell depletion. *p*<0.001. **D.1.** Total RNA was isolated from tumors of PBS, NLGP, PBS-CD8 depleted group (PBS-CD8 dep) and NLGP-CD8 depleted mice (NLGP-CD8-Dep) group (n = 3 in each case) to analyze genes, like, *cd31 and vegf* at transcriptional level by RT-PCR and **D.2.** densitometric analysis of band intensities from 3 individual observations (Mean ± SD) is presented. *p<0.001, **p<0.01. **D.3.** Immunohistochemical analysis of tumors obtained from PBS and NLGP pretreated mice with or without CD8 depletion were performed using monoclonal antibodies, specific for CD31, VEGF and VEGFR2.

Since, above experiments clearly indicate that immune system has a regulatory role in NLGP driven normalization of tumor angiogenesis, next, we studied the histological sections from carcinoma and melanoma tumors of NLGP and PBS pretreated mice. Prominent infiltration of immune cells was noticed in tumors from NLGP pretreated mice (Figure 4A.1). Flow cytometric analysis of cells from PBMC of either PBS or NLGP treated tumor bearing mice revealed increased CD8^+^ T cells in NLGP treated mice group (Figure 4A.2 and A.3). To validate the role of CD8^+^ T cells (if any), such cells were depleted using specific antibody, as work plan is schematically presented in Figure 4B.1. CD8^+^ T cell depletion was confirmed flow cytometrically (Figure 4B.2).

As we observed significant enhancement of CD8^+^ T cells within tumors from NLGP pretreated mice, we wanted to decipher the contributing role of these effector cells in NLGP mediated normalization of angiogenesis by *in vivo* depletion of CD8^+^ T cells as described in Materials and Methods and Fig. 4B.1. When mice were sacrificed on day 28 post B16F10 tumor inoculation, analysis of angiogenesis at that time point clearly suggested a predominant role for CD8^+^ T cells in NLGP driven immune-mediated vascular normalization, since CD8^+^ T cell depletion completely abolished anti-angiogenic potential of NLGP pretreatment (Figure 4C). NLGP mediated downregulation of VEGF, VEGFR2 and CD31 was again upregulated in mice group with CD8^+^ T cell depletion, as indicated by RT-PCR (Figure 4D.1, D.2) and immunohistochemical (Figure 4D.3) analysis.

### NLGP mediated vascular normalization is not due to the T cell mediated apoptosis of CD31^+^ cells rather due to unavailability of VEGF

**Figure 5:**
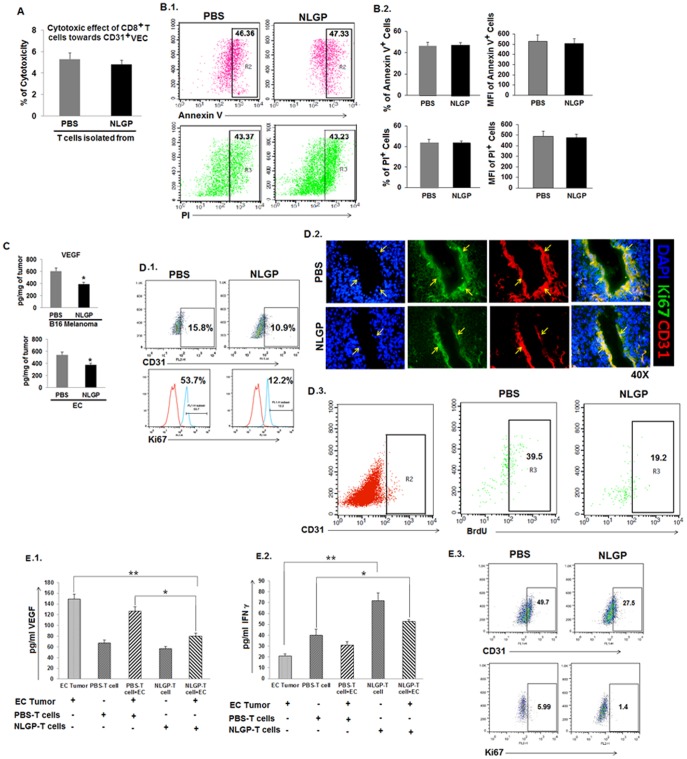
NLGP mediated vascular normalization is not due to CD8^+^ T cell mediated apoptosis of CD31^+^ cells. Mice were inoculated with B16 melanoma cells (2×10^5^ cells/mice) to grow tumor. After reaching the tumor volume to a considerable size (1372 mm^3^ approximately), tumor was harvested and CD31^+^ VECs were isolated by flow sorting. CD8^+^ T lymphocytes were isolated from NLGP or PBS pretreated (4×) mice by MACS purification and CD8^+^ T cells were co-cultured with the CD31^+^ VECs. **A.** Cytotoxicity was measured by LDH release assay. NLGP pretreated C57BL/6 mice were inoculated with B16 melanoma cells as mentioned earlier. **B.1.** As tumor reached a considerable volume (1372 mm^3^ approximately), tumors were harvested, single cells prepared and stained with anti-CD31 antibody along with either Annexin V or Propidium Iodide (PI)**.** Representative figures of Annexin-V and PI^+^ cells from CD31 gated population. **B.2.** Bar diagram showing % positive cells and MFI. **C.** Cell lysates prepared from carcinoma and melanoma tumors of PBS and NLGP pretreated mice were used to quantitate the level of VEGF by ELISA. Cytokines were measured as pg/mg of tissue ± SE and Mean ± SD of 3 individual observations are presented in bar diagram. *p<0.01. **D.1.** Obtained cells as mentioned in **B.1**, were stained for CD31, along with Ki67. Gated CD31^+^ population was assessed for Ki67 staining using Flowjo software and presented in histogram. **D.2.** Cryo-sections obtained from tumors of NLGP and PBS pretreated mice were stained with fluorescence labeled anti-CD31 (red) and anti-Ki67 (green) antibodies, along with DAPI (blue). Representative figures from 3 separate sets of experiments are presented. **D.3.** PBS and NLGP pretreated tumor bearing mice were injected with BrdU within tumor and sacrificed after 48 hours. Single cells were prepared to check BrdU staining after gating the CD31 population, as shown in a representative figure. **E.1, E.2.** PBMC were isolated from both PBS and NLGP treated tumor bearing mice and cultured with EC cells (2×10^5^ cells) for 24 hours and cell free supernatant were measured in pg/ml for VEGF (E.1) and IFNγ (E.2) by ELISA. Cytokines were quantitated as pg/ml ± SE. *p<0.001, **p<0.01. **E.3.** Flow sorted CD31^+^ ECs isolated from tumor microenvironment were cultured with the above mentioned supernatants (NLGP-PBMC+EC vs PBS-PBMC+EC) for 48 hours and assessed for the EC proliferation flow cytometrically after Ki67 labeling.

Since CD8^+^ T cells are found to be responsible for anti-angiogenic effect of NLGP-conditioned immune system (preferentially achieved by downregulation of VEGR2^+^CD31^+^ endothelial cells), initially we analyzed direct cytolytic effect of CD8^+^ T cells on CD31^+^ VECs. CD31^+^ cells were flow sorted from solid B16F10 tumors (Figure S1) and exposed to CD8^+^ T cells from NLGP pretreated tumor bearing mice. Co-incubation study suggested that CD8^+^ T cells are unable to exert any direct cytolytic effect *in vitro* against tumor derived CD31^+^ endothelial cells (Figure 5A). Furthermore, analysis of pro-apoptotic and apoptotic/necrotic VECs using Annexin V and PI respectively within tumor revealed that NLGP pretreatment has no effect on early apoptosis or late apoptosis/necrosis of CD31^+^ cells (Figure 5B.1, B.2).

Analysing these two above mentioned results and considering the importance of VEGF as rate limiting factor for uncontrolled VEC proliferation and survival (necessary for neovascularization) next we assessed *in situ* VEGF concentration and its influence in NLGP mediated vascular normalization. Evidences obtained from ELISA clearly suggested that availability of VEGF is low in tumor *in situ* from NLGP pretreated mice (Figure 5C), which might regulate CD31^+^ endothelial cell proliferation. To further verify this possibility, CD31^+^Ki67^+^ proliferating cells were analyzed in B16F10 tumor from NLGP pretreated mice by flow cytometric (Figure 5D.1) and immunofluorescence analysis (Figure 5D.2). Consistently, *in vivo* BrdU labeling and analysis of CD31^+^BrdU^+^ proliferating cells within harvested cells from tumors indicated significant lowering of proliferating cells in tumor bearing mice pretreated with NLGP (Figure 5D.3). The obtained results clearly suggested that the presence of proliferating endothelial cells is significantly less in NLGP pretreated mice than control mice. Again, peripheral blood mononuclear cells (PBMC) from EC bearing PBS and NLGP treated mice were co-cultured with tumor (EC) cells and culture supernatant was analyzed for VEGF and IFNγ content. Analysis of such supernatants revealed low content of VEGF and high IFNγ (in NLGP-PBMC-EC cell co-culture), in comparison to those where PBS-PBMC was used (Figure 5E.1, E.2). Furthermore, flow-sorted CD31^+^ cells were *in vitro* exposed to supernatants from NLGP-PBMC-EC cell co-culture and proliferation (monitored by Ki67 staining) of CD31^+^ endothelial cells was monitored, where significantly less proliferation was noted due to supplementation of supernatant from NLGP-PBMC-EC cell co-culture (Figure 5E.3). Therefore, these results clearly pointed out the prominent role of VEGF downregulation caused after NLGP-instructed CD8^+^ T cell infiltration in the reciprocal regulation of VEC proliferation and vascular normalization.

### NLGP mediated vascular normalization has no adverse effect on normal wound healing process in mice

**Figure 6:**
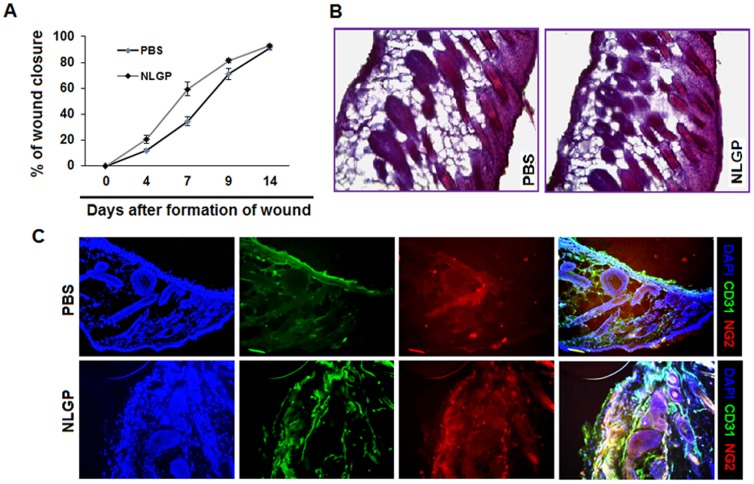
NLGP mediated vascular normalization has no adverse effect on normal wound healing process. Swiss mice were pretreated with NLGP (25µg) and PBS once in a week for four weeks and 4 mm^3^ wound was made on the back of both groups of mice. **A.** Diameter of wounds was measured every two days and percentages of wound closure were calculated and data presented as Mean ± SD of 6 individual observations. **B.** Histological sections of wound beds were stained with H&E and assessed microscopically. Representative figures are presented. **C.** Cryo-sections of wound beds were stained with fluorescence labeled anti-CD31 (red) and anti-NG2 (green) antibodies along with DAPI.

Given the potential safety concerns of systemic toxicity for strategies targeting tumor angiogenesis, we also intended to evaluate the impact of NLGP on cutaneous wound healing process (model of physiological angiogenesis). Mice were treated with NLGP or PBS as described earlier and 4 mm^2^ wounds were made in the skin of upper back. As shown in (Figure 6A) extent of wound closure in early days (between 4–7) was slightly higher in NLGP treated group, but in later stages (on day 9–14) healing was faster in PBS group and finally all wounds healed fully within a 2-week period. Furthermore, histological analysis revealed no observable differences in skin from both group of mice showing signature of wound healing (rapid epithelialization along with adipose layer were observed and hair follicles were formed) (Figure 6B). Immunofluorescence analysis on CD31^+^ and NG2^+^ cells on skin clearly showed no significant changes in wound healed skin from NLGP and PBS pretreated mice (Figure 6C). Therefore, our results clearly suggest that modulatory effect of NLGP on tumor angiogenesis does not hamper the normal physiological angiogenic process.

## Discussion

We have reported significant restriction of murine sarcoma, carcinoma and melanoma growth due to administration of NLP/NLGP in prophylactic [15], [16], [29] and therapeutic [17], [18] settings. This tumor growth restriction is strictly dependent upon modulation of host-tumor immune interaction [20], [25], since NLGP is unable to induce direct tumor cell apoptosis [19], [35]. Apart from the already discussed immunomodulation by NLGP in cancer [18], [20], [22], angiogenic normalization property of this molecule is described here for the first time.

As results suggest, prophylactic administration of NLGP (with an interval of 7 days for 4 times) is inhibitory towards neo-vascularization initiated after tumor challenge and the anti-angiogenic effect is indeed associated with the decrease in heavily dilated (thick)/fragile as well as very thin blood vessels. However, the thin and compact vessels were observed to be retained, probably to facilitate the trafficking of immune effector cells. Based on the available data, we reasoned that NLGP pretreatment causes significant reduction in proliferating Ki67^+^CD31^+^ VECs within tumor and thereby reduces the tumor micro vessel density (an indicator of tumor angiogenesis). Since proliferation of CD31^+^ cells corroborates neovascularization [36], [37], such reduction plays a great role in angiogenic normalization. Unlike VECs, NLGP do not decrease the number of NG2^+^ pericytes, but effectively preserve their maturity and coverage of these cells on blood vessels. Under NLGP influence, their tight association with VECs restore the vessel integrity and prevents leakiness. Therefore, by differentially regulating the two important stromal cell features, NLGP controls aberrant tumor vasculature. In context to host-antitumor benefits such results are encouraging as recent preclinical and clinical findings suggest that vascular normalization, rather than restriction of blood flow, is necessary to maintain the surge of effector immune cells and chemical regimens for cancer therapy [38].

Evidences are accumulated from present study suggesting the interference of NLGP in balancing tumor growth-supportive pro- and anti-angiogenic molecules. Several previous studies show that the VEGF family proteins, which signals through VEGFRs [39]–[41] are major factors involved in tumor-induced angiogenesis. Tumor and residing stromal cells secrete several growth factors particularly VEGF [42], [43] to stimulate VEGFR^+^ endothelial cell proliferation and in turn these cells provide the lining of newly formed blood vessels to supply nutrient to growing tumor [39]. Among all VEGFRs, VEGFR2 is mainly found on newly proliferating endothelial cells and targeting of VEGFR2 has been shown in some tumor models to reverse neo-vascularization [44]. Accordingly, NLGP selectively targets the VEGF-VEGFR2 signaling in proliferating endothelial cells to create a ‘vascular normalization window’ that might facilitate a decrease in interstitial pressure, enhanced tumor oxygenation and ultimately leads to a better therapeutic response [39], [40] in terms of restricted tumor growth [27].

In view of our consistent observation on central involvement of immune system in NLGP-mediated eradication or prevention of murine tumor growth [17]–[20], the present study additionally evaluated the involvement of NLGP-instructed immune-modulation in controlling tumor-angiogenesis. Interestingly, we observed a significant abolition of NLGP mediated both anti-angiogenic and anti-tumor effect in cyclosporine [45], [46] treated mice having prominent immunosuppression. However, adoptive transfer of immune cells from mice with NLGP therapy again restores both anti-angiogenic and tumor growth restricting effects of NLGP. Analysing these data, we speculated that NLGP-driven immune activation might be involved in anti-angiogenic process. To further validate our hypothesis, we used immunocompromised athymic nude mice and here also NLGP prophylaxis was unable to prevent neovascularization as well as tumor growth. Next, we directly focussed on the contribution of CD8^+^ effector T cells, since NLGP selectively increases the trafficking of these effector cells into tumor parenchyma and therapeutic NLGP mediated tumor growth restriction is abrogated completely in CD8^+^ T cell depleted mice [17], [18]. However, infiltrating CD8^+^ T cells often unable to show cytotoxic effect because, several tumor microenvironmental factors upregulate expression of inhibitory molecules like PD1 and CTLA4 on T cells to attenuate its effector functions and effector cytokine production [47]. In this context, modulatory effect of NLGP on TME is already reported [18], [27], [28]. More importantly, NLGP minimizes TME-induced anergy and exhaustion of CD8^+^ T cells, as observed by downregulation of anergy related molecules DGKa, Grail, EGRs etc. [18] and exhaustion related molecules TIM3, LAG3, PD1 and CTLA4 [17], [19] to preserve the optimum functional efficacy of infiltrated CD8^+^ T cells. Likewise, in present study, NLGP administration followed by CD8^+^ T cell depletion was unable to produce anti-angiogenic effect, as dilated tortuous (thick) blood vessels are seen in these groups of animals. Moreover, NLGP mediated reduction of proliferating CD31^+^ endothelial cells or VEGF-VEGFR2 expression within tumor is abrogated in CD8^+^ T cell depleted tumor bearing mice.

Considering this important contribution of CD8^+^ T cells in NLGP mediated anti-angiogenesis, initially we assumed that CD8^+^ T cells might be directly involved in killing of CD31^+^ VECs. In several previous studies, it was demonstrated that VEGFR2-specific CTL can be directly involved in the killing of proliferating VECs [48]. Contrary to these reports in our system we do not observe any cytolytic activity of CD8^+^ T cells isolated from NLGP treated mice towards flow sorted CD31^+^ VECs. To solve this puzzle, we checked involvement of VEGF, for which sorted CD31^+^ VECs were incubated with supernatants from co-culture of PBMC from NLGP/PBS EC bearing mice and EC cells. Interestingly, CD31^+^ VECs proliferated less with NLGP-PBMC+EC cells culture supernatant (having comparatively high level of VEGF), which was again compensated with addition of recombinant VEGF. *In vivo* BrdU labeling study also suggests less number of CD31^+^BrdU^+^ proliferating cells within tumors from NLGP pretreated mice, where VEGF content is significantly less. Therefore, finally, we concluded that unavailability of VEGF might be the predominant rate limiting factor of reduced VEC growth and vascular normalization. Considering the infiltration and essential role of CD8^+^ T cells, we further assumed that, NLGP treatment may enhance DC migration to lymph node to prime CD8^+^ T cells, which eventually infiltrates tumor parenchyma to kill tumor cells (that serves as one of the prime source for VEGF). It could also be possible that infiltrated CD8^+^ T cells produce IFNγ and/or infiltrated DC produce IL-12 and either of these immunomodulatory cytokines possess anti-angiogenic effects by altering pro-angiogenic mediators [29], [49]. Interestingly, our previous studies suggested the effectiveness of NLGP to influence CD8^+^ T cells and DC to produce IFNγ and IL-12 respectively [20]–[22]. On the other-hand, in a separate *in vitro* study, we observed that NLGP can directly modulate B16 melanoma tumor cells by reducing HIF1α and VEGF in normoxic as well in hypoxic condition (*unpublished observation*).

In summary, our results suggest that NLGP prophylaxis educate whole immune system in such a way that after tumor challenge antigen presenting cells efficiently prime effector CD8^+^ T cells, which in due course kill tumor cells to reduce tumor promoting growth factor burden within TME. These reduced availability of growth factor especially VEGF subsequently impede the growth of endothelial cells without affecting the vessel integrity to maintain the proper trafficking of immune effector cells within TME. More importantly this strategy does not affect the normal wound healing process, since immune-elimination is not obligate here. However, whether these infiltrated CD8^+^ T cells affect other VEGF producing cells or any other parallel cascade operational in this NLGP-instructed immune system-mediated anti-angiogenic process needs further evaluation. Considering the limitation of anti-angiogenic immunotherapy [50], [51] combination therapy integrating anti-angiogenic therapy along with immunotherapy or other conventional therapy was proposed by several groups [52], [53]. In this context, NLGP treatment would be more promising in the field of cancer management because of its multidirectional fine tuning ability of tumor vasculature as well as of systemic/local immunity without any adverse physiological consequence.

## Methods

### Ethics statement on mice experiments

For maintenance and experimentation on mice, the relevant guidelines were followed and the Chittaranjan National Cancer Institute animal ethical committee approved the study.

### Mice and tumors

Female C57BL/6 and Swiss mice (Age: 4–6 weeks; Body weight: 24–27 g) were obtained from the National Centre for Laboratory Animal Sciences (NCLAS), Hyderabad and Institutional Animal Care and Maintenance Department, Chittaranjan National Cancer Institute (CNCI), Kolkata, India respectively and maintained under standard laboratory conditions. Immuno-compromised athymic nude mice (4–6 weeks old) were purchased from NCLAS, Hyderabad and maintained in a specific pathogen free facility. Autoclaved dry pellet diet (Epic Laboratory Animal Feed, West Bengal Govt, Kalyani, India) and water were supplied *ad libitum*. Ehrlich Carcinoma (EC) was maintained by regular *in vivo* intraperitoneal passage in Swiss mice. B16F10 melanoma cell line was cultured *in vitro* in DMEM supplemented with 10% (v/v) FBS, 2 mM L-glutamine and penicillin-streptomycin (100µg/ml) at 37°C humidified conditions. To develop solid tumors *in vivo*, C57BL/6 and Swiss mice were inoculated subcutaneously (s.c.) in right hind leg quarters with B16F10 melanoma cells (2×10^5^) and EC cells (1×10^6^) respectively.

### Antibodies and reagents

RPMI 1640, DMEM, and FBS were purchased from Invitrogen (NY, USA). Lymphocyte separation media (LSM) was procured from MP Biomedicals, Irvine, CA, USA and HiMedia, Mumbai, India. Fluorescence conjugated different anti-mouse antibodies (CD4, CD8, Ki67) and purified CD31 were procured from either BD-Pharmingen or Biolegends (*both in,* San Diego, CA, USA). Fluorescence- or peroxidase-labeled secondary antibodies were procured from e-Biosciences (San Diego, CA, USA). Purified anti-mouse Foxp3, VEGF, VEGFR1, VEGFR2, were procured from Santa Cruz Biotech (California, USA). IFNγ/IL-10 estimation kits (OptEIA, BD Biosciences, San Jose, CA, USA) 3,3′,5,5′-tetramethylbenzidine (TMB) substrate solutions (for ELISA), CytoFix/CytoPerm kit (for intracellular staining), AnnexinV-Propidium iodide apoptosis detection kit were obtained from BD Pharmingen, San Dieago, CA, USA. LDH release assay kit for cytotoxicity and BrdU kit for proliferation were obtained from Roche Diagnostics, Mannheim, Germany. Western lightining chemiluminescence and immunoperoxidase color detection kit were purchased from Pierce (Rockford, IL, USA) and Vector laboratories Inc (Burlingame, CA, USA) respectively. Optimal cutting temperature (OCT) compound was purchased from Sakura Finetek, Torrance, CA, USA. RT-PCR primers were procured from MWG-Biotech AG (Bangalore, India). DAPI was purchased from Sigma, St. Louis, MO, USA.

### Neem leaf glycoprotein

Mature neem *(Azadirachta indica)* leaves of identical size and color (indicative of similar age), taken from a standard source were shed-dried and pulverized. Leaf powder was soaked overnight in phosphate buffered saline (PBS), pH 7.4 and supernatant was collected by centrifugation at 1500 rpm, termed neem leaf preparation (NLP) [16], [54]. NLP was then extensively dialyzed against PBS and concentrated by Centricon Membrane Filter (Millipore Corporation, Bedford, MA, USA) with 10 KDa molecular weight cut off. Active component of this preparation is a glycoprotein, as characterized earlier [55] and designated as Neem leaf glycoprotein (NLGP). Protein concentration of NLGP solution was measured by Lowry's method [56] using Folin's Phenol reagent. Purity of the NLGP was confirmed by HPLC [22] before use.

### NLGP injection and tumor growth restriction assay

Two groups (n = 8, in each group) of either C57BL/6 or Swiss mice were immunized once weekly (25µg/100µl PBS/mice s.c.) for 4 weeks in total at left hind leg quarter with NLGP, keeping other group as PBS control. Immunized mice were inoculated with B16F10 and EC tumors respectively as mentioned above to develop solid tumors. Growth of solid tumor (in mm^3^) was monitored biweekly by caliper measurement using the formula: (width^2^×length)/2. Survival of mice was noted regularly, till tumor size reached to 25 mm in either direction.

### Angiogenesis study with blood vessels

To study the role of NLGP on tumor angiogenesis, both groups of NLGP and PBS pre-treated tumor bearing mice were sacrificed and skin were removed carefully from peritoneal region without disturbing the angiogenic vessels adjacent to tumors. These blood vessels were counted macroscopically using convex lens depending on the thickness of the blood vessels and categorized as heavy, very thick, thick, thin and very thin. Area of blood vessels was calculated using Photoshop software (Adobe Systems Incorporated, San Jose, California, USA) and presented in Pixels. Extent of angiogenesis was categorized as 4 (++++), 3 (+++), 2 (++) and 1 (+). Tumor volume (in mm^3^) and extent of angiogenesis (in raw score) was multiplied to obtain an index for tumor angiogenesis. Mean of score from all mice was presented as Mean Index for Tumor and Angiogenesis (MITA).

### Angiogenesis in immunocompromised mice

To study the role of immune system in tumor angiogenesis, Swiss mice were divided into three groups (n = 3) and two groups received NLGP immunization as said before, keeping other group as PBS control. One of these NLGP treated mice group was immune suppressed by three consecutive peritoneal cyclosporine injections (15 mg/Kg) on day 13, 17 and 21. Mice of all groups were inoculated s.c. with EC (1×10^6^ cells) on day 24. Again, cyclosporine was injected on day 25 and 28. Similar study was conducted on immune compromised athymic nude mice, where one group received NLGP with another PBS control group. Following completion of immunization all mice received EC (1×10^6^ cells) s.c. and tumor growth and survivability were monitored biweekly. Pattern of angiogenesis was noted after sacrificing the mice.

To reconfirm the same, NLGP immunized Swiss mice were similarly divided in three groups (n = 3, in each group) and immunologically suppressed with consecutive peritoneal injection of cyclosporine as mentioned above. Then all three groups of mice were injected with 1×10^6^ viable EC cells. Following establishment of tumor (64 mm^3^ in average), first group was kept as control, second group received splenic immune cells (1×10^7^) i.v. through tail vein from PBS treated mice and third group of mice received same number of immune cells from 4× NLGP (25µg/100µl/mice) immunized mice. When tumor reached a considerable volume (25 mm) in mice from PBS pretreated group, mice from both groups were sacrificed (on day 45) for comparative monitoring of angiogenesis, as described above. Identical experiment was performed in athymic nude mice with similar cell transfer from either PBS or NLGP injected mice.

### Angiogenesis in CD8^+^ T cell depleted mice

Within several immune cells, to study the specific role of CD8^+^ T cells in the process of angiogenesis, C57BL/6 mice were divided in four groups (n = 4 in each group). Two groups of mice were immunized with NLGP as said before while other two groups of mice were injected with PBS. One NLGP and one PBS treated mice group were peritoneally injected with CD8 depleting antibody (100µg/50µl) on day -1, 6, 13, 20 and 27 as shown in Fig. 4BI. CD8^+^ T cell depletion status was monitored regularly by analyzing peripheral blood using flow cytometry. On day 24, B16F10 tumors (2×10^5^ cells/mice) were inoculated s.c. to the left flank of hind leg. Tumor volumes were monitored biweekly and on reaching a considerable size (25 mm) in mice from PBS pretreated group, mice from both groups (PBS and NLGP) were sacrificed for comparative monitoring in angiogenesis, as described above.

### Tumor infiltrated immune cells

After attaining considerable size, tumors of either type were harvested from sacrificed mice. Portions of tumors were separately preserved for histology, immunohistochemistry, immunofluorescence studies, western blot, flow cytometry and RT-PCR analysis.

A piece of tumor was cleaned with PBS and chopped into small pieces and treated with mixture of collagenase (2µg/ml) and hyaluronidase (2µg/ml) and passed through the nylon mesh to prepare single cell suspensions. Tumor infiltrating lymphocytes (TILs) were then separated from tumor cells by differential gradient centrifugation at 2000 rpm for 30 minutes to analyze their proportions.

### Histology, immunohistochemistry and immunofluorescence studies

Tumors were fixed in 10% formalin for standard histological preparations and embedded in paraffin. Sections (4–5 µm) were prepared and stained with hematoxylin-eosin (H&E) according to standard protocol. Representative tumors were selected for immunohistochemical analysis. Fresh tumor tissues were also frozen for cryo-sectioning. Sections were immunostained for CD31, NG2, VEGF, VEGFR1 and VEGFR2 by the method described [27]. In some cases, tumor or skin sections were snap-frozen in OCT compound. Sections (5 µm) were prepared using cryostat (Leica, Germany), air-dried and fixed in ice-cold methanol for 20-30 min. The sections were blocked with 5% BSA solution and stained with different anti-mouse antibodies (CD31-PE, NG2-FITC, Ki67-FITC) by the method described earlier [27].

### Western blot analysis

Tumor lysate or cellular lysate (50 µg) were separated on 6–20% SDS–polyacrylamide gel and transferred onto a PVDF membrane for Western Blotting. Incubation was performed for different primary antibodies, e.g., CD31, NG2, VEGF, VEGFR1 and VEGFR2, and the procedure followed the method as published [18].

### RT-PCR analysis

Total RNA was isolated from solid tumors (from PBS and NLGP treated mice) using the TRIZOL Reagent (Ambion, Austin, Texas, USA). The cDNA synthesis was carried out using RevertAid First Strand cDNA Synthesis Kit (Fermentas, K1622) following the manufacturer's protocol and RT-PCR was carried out using gene-specific primers. The primer sequences of mouse CD31, VEGF, VEGFR1, VEGFR2, NG2 and β-Actin are described in the Table 2. PCR products were identified by image analysis software for gel documentation (Gel Doc XR+ system, BioRad) following electrophoresis on 1.5%–2% agarose gels and staining with ethidium bromide [18], [27].

**Table 2:** Primer sequences of various cytokine genes studied.

Name	Primer sequences (5′–3′)	Product size
β-Actin-forward	CAACCGTGAAAAGATGACCC	228 bp.
β-Actin-reverse	ATGAGGTAGTCTGTCAGGTC	
VEGFR2-forward	ACAGACAGTGGGATGGTCC	271 bp
VEGFR2-reverse	AAACAGGAGGTGAGCGCAG	
VEGFR1-forward	CCAACTACCTCAAGAGCAAAC	315 bp
VEGFR1-reverse	CCAGGTCCCGATGAATGCAC	
CD31-forword	AGCCCACCAGAGACATGGAA	337 bp
CD31-reverse	CTGGCTCTGTTGGAGGCTGT	
VEGF-forward	GGACCCTGGCTTTACTGCTG	201 bp
VEGF-reverse	CACAGGACGGCTTGAAGATG	

### Flow cytometric staining

Single cell preparation from harvested tumors were labeled with 0.5µl (for 1×10^6^ cells) FITC or PE conjugated antibodies, specific for mouse CD8, CD31, Ki67 markers, and surface or intracellular flow cytometry was performed by the method described [17].

### Annexin V-PI staining for apoptosis

Harvested tumors from PBS and NLGP treated mice were minced to make single cell suspension as mentioned before. Freshly collected single cells were mixed with 1× binding buffer (100 µl) and kept for 2 min at room temp. Then 5µl of each Annexin-V and PI were added and incubated for 15 mins and then finally analyzed by flow cytometry.

### Flow sorting of CD31^+^ cells

Single cell suspension obtained from harvested tumors was washed with PBS (containing 1% FBS) and passed through cell strainer. This cell pellet was stained with primary anti-mouse-CD31 antibody (30 minutes) and further tagged with appropriate FITC labeled secondary antibody and CD31^+^ cells were purified by Flow sorting with BD FACS Aria, San Jose, CA.

### Mechanistic studies on downregulation of CD31^+^ endothelial cells

CD8^+^ T cells were purified by MACS from the spleen of both PBS and NLGP treated tumor bearing mice by the method described [17], [18]. Flow sorted CD31^+^ endothelial cells were co-cultured with CD8^+^ T cells in 1∶10 dilution in serum free media and checked for cytotoxicity by LDH release assay. Cell-free supernatants were used to measure the level of released LDH using the formula: % Cytotoxicity  =  (Lysis from Effector-Target Mixture – Lysis from Effector only) – Spontaneous Lysis/(Maximum Lysis – Spontaneous Lysis) ×100.

In a separate experiment, splenic cells were purified from EC bearing PBS and NLGP treated mice and co-cultured with EC cells (10∶1 ratio) for 24 hrs. Cell free culture supernatants were collected and assessed for VEGF and IFNγ content by ELISA. Purified CD31^+^ endothelial cells were also incubated with such culture supernatants for 48 hrs and their proliferation was assessed by Ki67 staining by the method described [15]. In a parallel experiment NLGP (4×) pretreated mice were injected with anti-mouse BrdU antibody injected in tumor as per manufacturer's manual. After 48 hours of injection both groups of mice were sacrificed to harvest tumors and single cells were prepared as described before. Single cells were stained with anti-CD31 antibody and assessed flow cytometrically as per standard protocol.

### Wound healing assay

Mice were pretreated with PBS and NLGP as described earlier. Mice were then anesthetized with peritoneal injection of 0.3 ml of 2-2-2-tribromoethanol (Avertin, Sigma, St. Louis, MO) and back portion was properly shaved to remove all fur and cleaned with 70% alcohol. Subsequently using dual puncher 4 mm^2^ wound was created on both side of their back and kept in sterile environment. After every 3 days interval wound closure was measured henceforth by a vernier caliper and the wound healing was analyzed. Finally on day 15 mice of both groups were sacrificed and their skins were fixed and sectioned using cryostat. Routine histology and immunofluorescence study was performed in skins.

### Statistical analysis

All results represent the average of separate *in vivo* and *in vitro* experiments. Number of experiments is mentioned in result section and legends to figures. In each experiment a value represents the mean of three individual observations and presented as mean ± standard deviation (SD). Statistical significance was established by Student's t-test using INSTAT 3 Software (GraphPad Software, Inc.), with differences between groups attaining a *p* value <0.05 considered as significant.

## Supporting Information

Figure S1 **Purification of CD31^+^ cells by flow sorting.** Solid B16 melanoma tumors were harvested from PBS treated C57BL/6 mice and single cell preparation was made. Cells were labeled with anti-CD31 antibody and positive cells were sorted in flow cytometer (BD FACS Aria). A. FSC/SSC plot of single cell population under study. B. Unstained cell population in FL1 (CD31)/FSC plot. C. CD31^+^ cells in FL1 (CD31)/FSC plot. D. Purified CD31^+^ vascular endothelial cells after flow sorting.
(TIF)
