# Supplementary figures and images for: Neem Leaf Glycoprotein Prophylaxis Transduces Immune Dependent Stop Signal for Tumor Angiogenic Switch within Tumor Microenvironment
Source: PLoS One. 2014 Nov 12;9(11):e110040. doi: 10.1371/journal.pone.0110040 (PMC4229107; doi:10.1371/journal.pone.0110040)

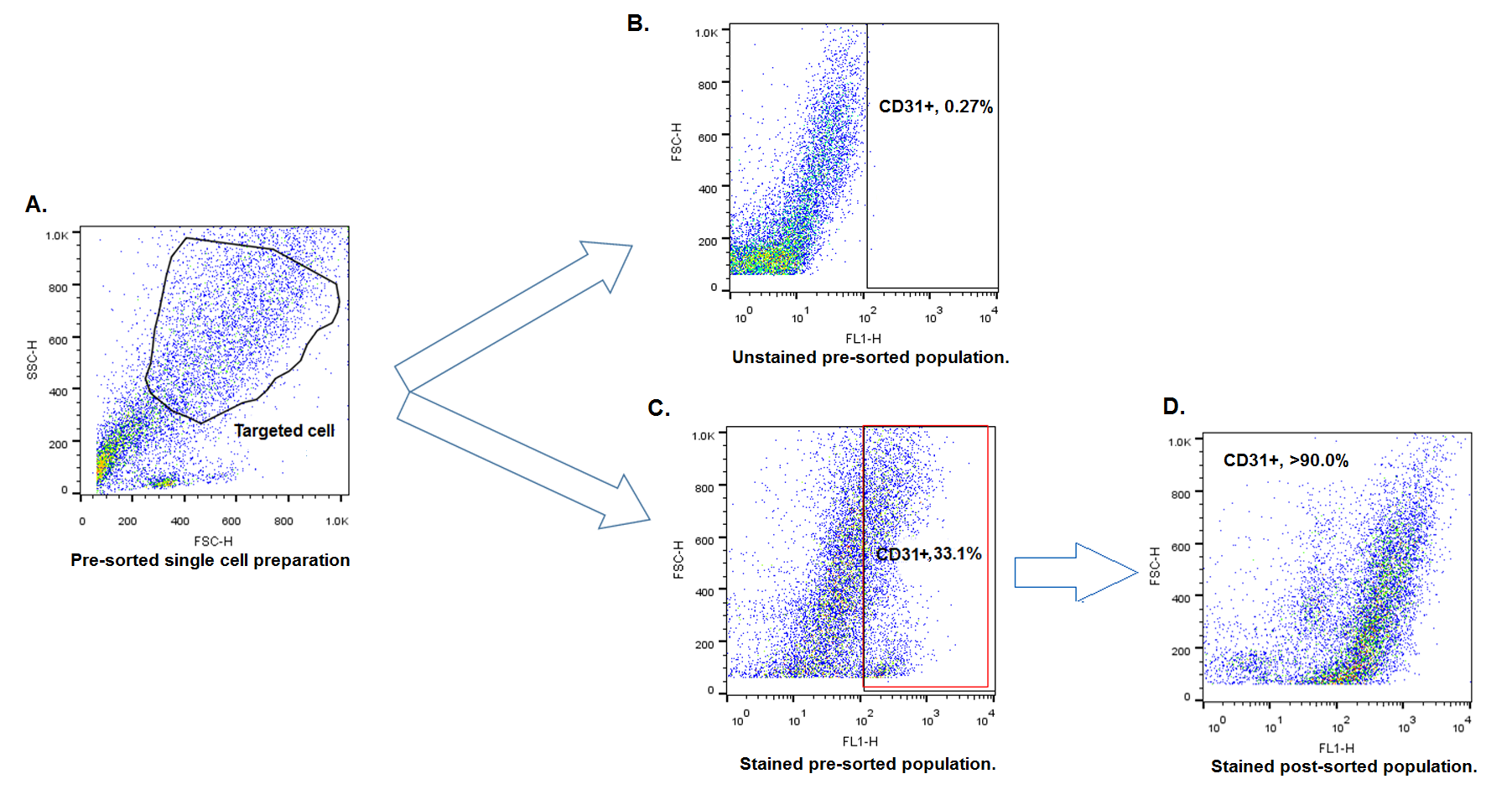

Supplement: Figure S1 — Purification of CD31+ cells by flow sorting. Solid B16 melanoma tumors were harvested from PBS treated C57BL/6 mice and single cell preparation was made. Cells were labeled with anti-CD31 antibody and positive cells were sorted in flow cytometer (BD FACS Aria). A. FSC/SSC plot of single cell population under study. B. Unstained cell population in FL1 (CD31)/FSC plot. C. CD31+ cells in FL1 (CD31)/FSC plot. D. Purified CD31+ vascular endothelial cells after flow sorting. (TIF) [file pone.0110040.s001.tif]
